# Accuracy of Visual Inspection Alone to Assess Joint Effusions of the Hand: Cross-Sectional Study

**DOI:** 10.2196/86261

**Published:** 2026-04-16

**Authors:** Carrie Ye, J Ross Mitchell, Shahad Al-Matar, Mena Bishay, Niall Jones, Dalton Sholter, Dylan Johnson, Steven Katz, Anthony S Russell, Mohamed Abdalla

**Affiliations:** 1 Department of Medicine, Faculty of Medicine & Dentistry, University of Alberta, 8-130 Clinical Sciences Building, 11350 83 Ave NW, Edmonton, AB, T6G2G3, Canada, 1 780-492-7002, 1 780-492-6088; 2 Arthritis Research Canada, Vancouver, BC, Canada; 3 Alberta Machine Intelligence Institute, Edmonton, AB, Canada

**Keywords:** arthritis, rheumatoid arthritis, effusions, telehealth, rheumatology

## Abstract

**Background:**

Physical examination is the cornerstone of diagnosing and monitoring inflammatory arthritis, with the detection of joint effusions being one of the most crucial components of the examination. Rheumatologists largely rely on palpation, supported by other examination techniques, such as evaluating the range of motion and visual inspection, to assess signs of joint swelling. However, remote care is on the rise in rheumatology, and aside from visual inspection, physical examination of joints is limited during telehealth visits. The ability of rheumatologists to accurately detect hand synovitis through photos or videos of the hands, without the benefit of direct tactile examination, is currently unknown.

**Objective:**

This study aimed to assess the accuracy of detecting joint effusions of the hands by remote visual evaluation alone.

**Methods:**

We conducted a prospective cohort study of patients assessed by a rheumatologist in Edmonton, Alberta. Participants were assessed clinically by rheumatologists for the presence of effusions of the metacarpophalangeal (MCP) and proximal interphalangeal (PIP) joints between June 13, 2024, and October 29, 2024. Participants had photos and videos of their hands taken by study staff on the same day. Photos and videos were remotely assessed separately by 4 rheumatologists not involved in the initial clinical assessments for the presence of MCP and PIP joint effusions and compared with the clinical assessments.

**Results:**

The study cohort included 156 patients (mean age 53, SD 14.2 years, n=105, 67.3% were female and n=52, 33.3% had rheumatoid arthritis), for a total of 3120 MCP and PIP joints. Effusions were identified in 12.8% (20/156) of patients and in 2.2% (69/3120) of joints per clinical assessment. The average visual assessment joint-level sensitivity and specificity of photos were 0.14 (95% CI 0.07‐0.19) and 0.97 (95% CI 0.96‐0.98), respectively. The average visual assessment joint-level sensitivity and specificity of videos were 0.24 (95% CI 0.13‐0.33) and 0.98 (95% CI 0.97‐0.99), respectively. The average person-level visual assessment sensitivity and specificity of photos were 0.44 (95% CI 0.30‐0.60) and 0.82 (95% CI 0.76‐0.87), respectively. The average person-level visual assessment sensitivity and specificity of videos were 0.48 (95% CI 0.35‐0.60) and 0.84 (95% CI 0.79‐0.89), respectively. Assessor agreement was poor (κ=0.12‐0.17).

**Conclusions:**

Visual inspection of photos and videos to detect MCP and PIP joint effusions was poor at both the joint and person levels. Patients and rheumatologists should be aware of these limitations when conducting remote telehealth assessments.

## Introduction

Rheumatic diseases include a wide range of diagnoses that affect the musculoskeletal system and, according to the World Health Organization, are the leading cause of worldwide disability [1]. Access to specialized rheumatology care remains a significant challenge for many individuals, with female patients and those residing in smaller centers experiencing longer wait times [2,3]. In many cases, patients face long wait times and limited availability of specialists, often resulting in delays in diagnosis and treatment, which can lead to irreparable damage [4]. The Arthritis Society of Canada recently released *The State of Arthritis in Canada Report Card*, which scored all provinces and territories a grade of C or lower (ie, satisfactory, improvement needed, or significant improvement needed) for access to care, resulting in their top recommendation: “Improve access to arthritis care” [5]. The shortage of trained rheumatologists and the concentration of services in urban centers exacerbate the inequities in access to rheumatology care [5,6]. These barriers can lead to underdiagnosis and inadequate management of conditions such as rheumatoid arthritis, where timely intervention is crucial for preventing joint damage and improving patient outcomes [6-8].

In recent years, the shift toward remote care across medical specialties, including rheumatology, has gained momentum and was accelerated by the COVID-19 pandemic [9,10]. In March 2020, there was a 75.9% decrease in outpatient office visits, with nearly all visits completed by telemedicine, and by September 2021, half of patient encounters remained telemedicine visits [11]. The transition to telehealth appointments has provided a potential solution to address some of the access challenges in rheumatology [12]. An American study found that implementation of electronic consults in medical specialties led to a significant increase in the completion rate of specialist visits and a reduction in wait times [12]. Remote rheumatologist visits offer greater flexibility for patients who are unable to travel to rheumatology clinics. This model of care has the potential to improve health care equity by bridging geographical, economic, and gender gaps, particularly for individuals in underserved regions, those who cannot take time off of work to travel to appointments, or those who face childcare issues [2,13].

Traditionally, physical examination has been the cornerstone of diagnosing joint effusions and other musculoskeletal abnormalities in rheumatology [14]. Rheumatologists largely rely on palpation, supported by other examination techniques, such as evaluating the range of motion and visual inspection, to assess for signs of joint swelling [15]. However, aside from visual inspection, physical examination of joints is limited during telehealth visits. A recent scoping review found several studies that assessed the performance of unassisted physical examinations conducted over telehealth platforms, but no studies examined this from a rheumatology perspective, and it remains unclear how effective visual inspection alone is at detecting joint effusions during telehealth appointments [16]. The ability of rheumatologists to accurately detect hand synovitis, in particular, through photos or videos, without the benefit of direct tactile examination, is a critical question that has not been studied.

The objective of this study was to assess the accuracy with which rheumatologists can remotely detect hand joint effusions through images and videos of patients’ hands. By understanding the limitations and potential of remote visual assessment alone, this research seeks to inform best practices for telehealth in rheumatology and ultimately enhance patient care in a more equitable and accessible manner.

## Methods

### Study Design

We conducted a prospective cohort study at 4 rheumatology practices in Edmonton, Alberta. Participants were assessed by rheumatologists in the clinic for the presence of effusions of the metacarpophalangeal (MCP) and proximal interphalangeal (PIP) joints. After providing individual written consent, participants filled out a short intake survey on baseline characteristics and had photos and videos of their hands taken by a member of the research team on the same day. Photos and videos were assessed separately by rheumatologists not involved in the initial clinic assessments.

### Ethical Considerations

This study was approved by the research ethics board at the University of Alberta (Pro00141099). Informed consent was obtained for each participant. Each participant was assigned a unique study ID, and all study data were collected under this unique study ID, separate from the dataset that linked the study ID with the participant’s identifying information obtained in the consent form. Participants did not receive any compensation.

### Participants

Patients were recruited consecutively from 4 rheumatology clinics between June 13, 2024, and October 29, 2024. The inclusion criteria were being aged 18 years or older at the time of clinic appointment and being able to provide written consent. Patients with and without inflammatory arthritis were included. There were no exclusion criteria. Participants self-reported age, sex, diagnosis, duration of joint symptoms, height, and weight.

### Images and Videos

Photos and videos were taken using an 8 MP digital camera on a light blue background. A total of four photos were taken of both hands: (1) palms down, fingers spread; (2) palms down, fists; (3) palms up, fingers spread; and (4) palms up, fists. A single video including both hands was taken of each participant. For the video, each participant was instructed to have their palms down, spread out their fingers, and then make a fist 3 times. They were then instructed to repeat these movements with the palms facing up. Photos and videos included a few centimeters proximal to the wrists, ensuring that all fingertips were included in the images.

### Assessment of Joint Effusions

The reference standard labeling of joint effusions, defined as the accumulation of excess fluid in the joint cavity, was completed by the participant’s rheumatologist, who assessed each MCP and PIP joint during the physical examination portion of their appointment. All MCP and PIP joints had to be assessed by the rheumatologists, but physical examination techniques were not standardized between rheumatologists. Distal interphalangeal joint findings were not obtained, as these joints are rarely involved in inflammatory arthritis, and we wished to minimize the time burden on physicians. Rheumatologists labeled each joint as positive or negative for MCP or PIP joint effusion and were instructed to label uncertain joints as negative. Each participant was assessed by 1 rheumatologist. The rheumatologist was also asked to report whether there were visible deformities in the hands.

Asynchronously, 4 separate rheumatologists of varying experience (assessor 1: 1 year; assessor 2: 8 years; assessor 3: 15 years; and assessor 4: 50 years) were asked to assess the photos and videos on a computer screen (unlinked to participants and blinded to additional clinical information) for the presence of any MCP or PIP joint effusions (person-level assessment). If they felt there was MCP or PIP joint effusion present in any individual, they were asked to indicate the exact MCP and/or PIP joints in which they saw effusion (index test). Each evaluator was instructed to label each MCP and PIP joint as 0 (no effusion) or 1 (effusion) and to label uncertain cases as 0. No other specific training was provided to evaluators. Evaluators were allowed to view the images as many times and replay the videos as many times as they wished.

### Analysis

The sample size required to achieve a sensitivity of 0.90 with a lower bound of 0.80, power of 0.8, and alpha of .05, with an estimated prevalence of 5% effused joints, was 2140 joints or 107 individuals (20 joints per individual) [17]. For the same parameters, but for specificity, a sample size of 113 was required. To ensure an adequate sample size accounting for missing data, we targeted approximately 150 participants. Summary statistics were used to describe the baseline characteristics of the study cohort and the reference standard physical examination assessment of the MCP and PIP joints. Missing data were handled by available case analysis. Skin tone was assessed from the hand images by a member of the study team (MM) and categorized using a simplified Fitzpatrick skin scale (type 1 or 2=palest, type 3 or 4=medium, and type 5 or 6=darkest) [18]. Visual assessment of the photos and videos (index test) was compared at the joint and person levels with the reference standard assessment and reported as sensitivity (true positives/[true positives+false negatives]), specificity (true negatives/[true negatives+false positives]), positive predictive value (PPV; PPV=true positives/[true positives+false positives]), and negative predictive value (NPV; NPV=true negatives/[true negatives+false negatives]).

Agreement between the 4 evaluators was calculated using Fleiss kappa and pairwise comparisons between evaluators were calculated using Cohen kappa. Adequate visual performance was defined as concordance between physical examination and visual inspection by most of the assessors (at least 3 of 4 assessors). Baseline patient characteristics were compared for association with evaluator performance using chi-square tests. Correlation between assessor’s years of experience in rheumatology and performance (sensitivity and specificity) was assessed using the Pearson correlation coefficient. A *P* value of <.05 was considered statistically significant. All analyses were conducted using Python 3.9 (Python Software Foundation) using the *pandas*, *scikit-learn*, and *statsmodels* packages.

## Results

A total of 156 participants were recruited, with an average age of 53.2 (SD 14.2) years (Table 1). Of them, 105 (67.3%) were female. Most (n=90, 57.7%) participants had Fitzpatrick skin types 1 and 2. The most common rheumatologic diagnosis was rheumatoid arthritis (n=52, 33.3%), with an average duration of joint symptoms of 10 years. The average BMI was in the overweight category (29 kg/m^2^). Only 6 (3.8%) participants had notable joint deformities.

**Table 1.** Participant characteristics (n=156).

Characteristics	Participants
Age (y), mean (SD)	53.2 (14.2)
Sex, n (%)	
Female	105 (67.3)
Male	51 (32.7)
Skin tone, n (%)	
Fitzpatrick 1 and 2	90 (57.7)
Fitzpatrick 3 and 4	52 (33.3)
Fitzpatrick 5 and 6	13 (8.3)
Diagnosis, n (%)	
Rheumatoid arthritis	52 (33.3)
Psoriatic arthritis	21 (13.5)
Osteoarthritis	7 (4.5)
No known joint disease	24 (15.4)
Other	52 (33.3)
Duration of joint symptoms (years), mean (SD)	9.8 (10.1)
BMI (kg/m^2^), mean (SD)	29.2 (6.1)
Notable joint deformities, n (%)	6 (3.8)

Of the 156 individuals, 20 (12.8%) had joint effusion in at least one MCP or PIP joint, as assessed by their rheumatologist on physical examination (reference standard; Table 2). On average, there were 3.4 (SD 2.3) effused joints per individual assessed to have joint effusion. In total, 3120 MCP and PIP joints were assessed, with 69 (2.2%) joints found to have effusion per physical examination. Of these, 43 (1.4%) were MCP joints, and 26 (0.8%) were PIP joints.

**Table 2.** Rheumatologist physical examination findings (reference standard).

Analysis	Values
Person level (n=156)	
Joint effusion present, n (%)	20 (12.8)
Effused joints or an individual with joint effusion, mean (SD)	3.4 (2.3)
Joint level (n=3120), n (%)	
Joint effusion present	69 (2.2)
MCP^a^ joints	43 (1.4)
PIP^b^ joints	26 (0.8)

a MCP: metacarpophalangeal.

b PIP: proximal interphalangeal.

All 156 participants had photos, and 150 (96.1%) participants had videos. Individual assessor’s performance on photos and videos is listed in Tables S1 to S4 in Multimedia Appendix 1. Confusion matrices summarizing the overall group performance, using the threshold of at least 3 of 4 assessors labeling the presence of joint effusion being considered a positive index test, are included in Figure S1 in Multimedia Appendix 1. At the joint level, the average sensitivity was 0.14 (95% CI 0.07‐0.19) for photos and 0.24 (95% CI 0.13‐0.33) for videos, with a PPV of 0.14 (95% CI 0.06‐0.19) and 0.33 (95% CI 0.15‐0.37), respectively (Table 3). The average specificity was 0.97 (95% CI 0.96‐0.98) for photos and 0.98 (95% CI 0.97‐0.99) for videos, with an NPV of 0.98 (95% CI 0.98‐0.98) for both photos and videos. The kappa among all 4 evaluators was 0.12 (95% CI 0.07‐0.16) for photos and 0.17 (95% CI 0.11‐0.23) for videos.

**Table 3.** Accuracy of visual assessment at the person and joint levels, by image type.

Analysis	Photos, mean (95% CI)	Videos, mean (95% CI)
Joint level^a^		
Sensitivity	0.14 (0.07-0.19)	0.24 (0.13-0.33)
Specificity	0.97 (0.96-0.98)	0.98 (0.97-0.99)
PPV^b^	0.14 (0.06-0.19)	0.33 (0.15-0.37)
NPV^c^	0.98 (0.98-0.98)	0.98 (0.98-0.98)
Agreement^d^	0.12 (0.07-0.16)	0.17 (0.11-0.23)
Person level^e^		
Sensitivity	0.44 (0.30-0.60)	0.48 (0.35-0.60)
Specificity	0.82 (0.76-0.87)	0.84 (0.79-0.89)
PPV	0.31 (0.17-0.35)	0.38 (0.24-0.43)
NPV	0.91 (0.88-0.93)	0.92 (0.89-0.93)
Agreement^d^	0.17 (0.07-0.28)	0.17 (0.04-0.28)

a Photos: n=3120; videos: n=3000.

b PPV: positive predictive value.

c NPV: negative predictive value.

d Fleiss kappa between evaluators.

e Photos: n=156; videos: n=150.

The average sensitivity of visually detecting the presence of MCP or PIP joint effusion at the person level was 0.44 (95% CI 0.30‐0.60) from photos and 0.48 (95% CI 0.35‐0.60) from videos (Table 3), with a PPV of 0.31 (95% CI 0.17‐0.35) and 0.38 (95% CI 0.24‐0.43) for photos and videos, respectively. The average specificity was 0.82 (95% CI 0.76‐0.87) from photos and 0.84 (95% CI 0.79‐0.89) from videos, with an NPV of 0.91 (95% CI 0.88‐0.93) and 0.92 (95% CI 0.89‐0.93) for photos and videos, respectively. The kappa among all 4 evaluators was 0.17 (95% CI 0.07‐0.28) for photos and 0.17 (95% CI 0.04‐0.28) for videos. Pairwise agreements between assessors are listed in Tables S5 and S6 in Multimedia Appendix 1. Illustrative examples of cases with high and low agreement between assessors and concordant and discordant findings, compared with the reference standard, are shown in Figure S1 in Multimedia Appendix 1.

We did not find any patient characteristics that were statistically associated with performance by visual assessment (Table 4). We did not find a significant correlation between years of experience of the assessor and performance (sensitivity: correlation coefficient=0.29; *P*=.70, and specificity: correlation coefficient=0.07; *P*=.93).

**Table 4.** Patient characteristics associated with person-level performance^a^.

Characteristics	Photos (n=156)		Videos (n=150)	
	Adequate, n/N (%)	*P* value	Adequate, n/N (%)	*P* value
Age (y)		.89		.69
18‐39	26/32 (81.2)		29/32 (90.6)	
40‐69	74/99 (74.7)		76/92 (82.6)	
≥70	18/25 (72.0)		19/26 (73.1)	
Sex		.84		.70
Female	78/105 (74.3)		81/100 (81.0)	
Male	40/51 (78.4)		43/50 (86.0)	
Skin tone		.65		.91
Fitzpatrick 1 and 2	63/90 (70.0)		74/87 (85.1)	
Fitzpatrick 3 and 4	43/52 (82.7)		41/51 (80.4)	
Fitzpatrick 5 and 6	12/14 (85.7)		9/12 (75.0)	
BMI (kg/m^2^)		.70		.85
<30	71/97 (73.2)		77/94 (81.2)	
≥30	47/59 (79.7)		47/56 (83.9)	
Notable joint deformities present		>.99		.65
Yes	5/6 (83.3)		4/6 (66.7)	
No	113/150 (75.3)		120/144 (83.3)	

a Adequate performance was defined as concordance between physical examination and visual inspection by at least 3 of 4 assessors.

## Discussion

### Principal Findings

The results of this study demonstrated that rheumatologists were not able to accurately detect hand joint effusions through photos and videos, with the sensitivity being particularly low, which means that joint effusions of the MCPs and PIPs were missed by visual inspection alone. Specificity was higher for both photos and videos, suggesting that if joint effusions were found by visual inspection, they were likely present. Sensitivity and PPVs were slightly better at the person level than at the joint level, suggesting that visual inspection may be more appropriate to support triage and follow-up decisions than to assess disease activity. Agreement between assessors at both the person and joint levels was poor, underscoring the limitations of unaided visual inspection. Overall, performance was better with video assessment than with photo assessment, although these differences were not large. We were not able to detect any significant associations between patient characteristics and visual inspection accuracy, nor any correlation between assessors’ years of experience in rheumatology and visual inspection performance. These findings have important clinical implications, especially during remote telehealth visits.

The use of telehealth in rheumatology has been studied, but a recent review found that nearly all studies focused on patient and physician satisfaction, improving access to care, and reducing costs [13]. A recent scoping review of the unassisted physical examination conducted over telemedicine found 74 studies, with 8 that compared the telehealth musculoskeletal assessment to in-person assessment [16]. All these studies found that telehealth assessments were equivalent to in-person examination [19-26]. However, all except one of these studies were done in an orthopedic surgery setting, with one that was assessing Parkinson disease. None of these studies examined the performance of telehealth examination in the rheumatology setting. The low sensitivity observed in this study has important implications for telehealth in rheumatology. If joint effusions are missed during remote consultations, this could delay the detection of active inflammation, potentially leading to delays in treatment. Delay in diagnosis and treatment initiation for inflammatory arthritis can result in poor functional outcomes in patients [27]. There is also increasing evidence that treat-to-target strategies in rheumatoid arthritis improve patient outcomes; inaccurate assessment of swollen joint count and, therefore, disease activity can result in missed or delayed changes in therapy [28,29]. While telehealth appointments are necessary in certain situations and can improve access to specialty care for many patients, these limitations in visual examination for inflammatory arthritis need to be considered. The inability to fully capture all aspects of joint effusions through photos and videos may hinder the opportunity for timely therapeutic interventions, reducing the overall effectiveness of telehealth visits in managing these conditions. Moreover, most disease activity scores also require tender joint count, which can only be assessed through palpation [30].

One of the key strengths of this study is its use of a contemporary reference standard for comparison: the physical examination conducted by rheumatologists on the same day that the photos and videos were taken. This approach ensures that the findings are grounded in current clinical findings, providing a reliable benchmark for assessing the accuracy of photo and video assessments. Additionally, the study incorporated multiple assessors who were blinded to the patients’ clinical history and symptoms, minimizing potential bias and ensuring that the results reflect objective visual evaluation of joint effusions. The inclusion of a proportion of inflammatory joints that mirrors real-world clinical practice further strengthens the study’s relevance to everyday rheumatologic care.

### Limitations

A notable limitation of this study is the predominance of participants with lighter skin tones, limiting our ability to assess performance differences by skin tone. Skin tone may influence the visibility of joint swelling, and visual inspection may be less reliable in individuals with darker skin, potentially leading to differences in diagnostic accuracy. Moreover, the Fitzpatrick skin scale, while the most widely used and validated skin classification system, has not been specifically validated on hands and may perform less reliably when applied to images than in person [18,31]. Similarly, higher BMI and the presence of deformities could obscure the visibility of joint abnormalities, making it more difficult to accurately identify effusions during physical or remote assessments. However, likely due to insufficient sample size to assess these secondary outcomes, we did not detect differences in performance by these patient characteristics. We also did not collect data on the specific types of deformities noted; however, given the limited number of participants with deformities, we would not have been adequately powered to stratify our results based on the type of deformities present.

The prevalence of joint effusions was lower than that used for our sample size calculation, which would reduce the precision of our estimates. Fortunately, missing data were lower than expected, and thus, the recruitment of a larger sample size than calculated resulted in reasonable 95% CIs, suggesting stable estimates. Additionally, all images in this study were taken by someone on the research team, using the same camera, in a clinic setting. Real-life performance, particularly by patients, may vary by camera resolution, lighting, and user. While we used rheumatologists’ clinical examination as the reference standard in this study, the sensitivity of detecting clinical swelling in the hand joints by rheumatologists’ physical examination (compared with power Doppler ultrasound) is approximately 70% [32]. The addition of corresponding joint imaging would have allowed us to compare visual assessment to a more accurate reference standard and should be considered in future studies. Future studies could also examine the comparative and additive performance of patient-reported outcome measures compared with visual inspection for detecting active inflammatory arthritis [33].

The fact that rheumatologists are not able to accurately detect joint effusions through visual inspection signals promising opportunities for technological advancement. Artificial intelligence has been successfully trained to detect joint effusions of the hands using photos, although it has not been applied in a real-world setting [34]. Improving the reliability of remote assessments through artificial intelligence could make telehealth visits more effective, ultimately enhancing the management of active inflammatory arthritis and improving health care equity by ensuring that all patients, regardless of location and other sociodemographic factors, have access to accurate and timely care. However, the clinical collection and use of such large, representative datasets, particularly those containing sensitive patient images and videos, must be approached with caution and robust safeguards to ensure that patient privacy and data security are paramount.

In conclusion, remote visual inspection of joint effusions via photos and videos lacks the accuracy necessary for reliable detection, a limitation that must be carefully considered when conducting telehealth appointments.

## Supplementary material

10.2196/86261 Multimedia Appendix 1 Table S1-S4: Person- and joint-level performance of visual evaluation of photos and videos by individual assessor. Table S5: Person- and joint-level pairwise agreement for photos and videos. Figure S1 A-D: Confusion matrices for person- and joint-level photo and video overall assessor performance.

## Abbreviations

MCP: metacarpophalangeal
NPV: negative predictive value
PIP: proximal interphalangeal
PPV: positive predictive value
