# Supplementary material for: Accuracy of Visual Inspection Alone to Assess Joint Effusions of the Hand: Cross-Sectional Study
Source: J Med Internet Res. 2026 Apr 16;28:e86261. doi: 10.2196/86261 (PMC13085981; doi:10.2196/86261)
Supplement: Multimedia Appendix 1 [file jmir-v28-e86261-s001.docx]

**Supplementary Materials**

**Table S1. Person-level performance of visual evaluation of photos, by individual assessor**

| **Performance metric** | **Assessor** | | | |
| --- | --- | --- | --- | --- |
|  | **Assessor 1** | **Assessor 2** | **Assessor 3** | **Assessor 4** |
| **PPV** | 7/26 (0.27) | 7/14 (0.50) | 12/65 (0.18) | 9/31 (0.29) |
| **SEN** | 7/20 (0.35) | 7/20 (0.35) | 12/20 (0.60) | 9/20 (0.45) |
| **SPE** | 117/136 (0.86) | 129/136 (0.95) | 83/136 (0.61) | 114/136 (0.84) |
| **NPV** | 117/130 (0.90) | 129/142 (0.91) | 83/91 (0.91) | 114/125 (0.91) |

*PPV, positive predictive value; Sen, sensitivity; Spe, specificity; NPV, negative predictive value.*

**Table S2. Person-level performance of visual evaluation of videos by individual assessor**

| **Performance metric** | **Assessor** | | | |
| --- | --- | --- | --- | --- |
|  | **Assessor 1** | **Assessor 2** | **Assessor 3** | **Assessor 4** |
| **PPV** | 7/23 (0.30) | 9/15 (0.60) | 14/63 (0.22) | 8/19 (0.42) |
| **SEN** | 7/20 (0.35) | 9/20 (0.45) | 14/20 (0.70) | 8/20 (0.40) |
| **SPE** | 114/130 (0.88) | 124/130 (0.95) | 81/130 (0.62) | 119/130 (0.92) |
| **NPV** | 114/127 (0.90) | 124/135 (0.92) | 81/87 (0.93) | 119/131 (0.91) |

*PPV, positive predictive value; Sen, sensitivity; Spe, specificity; NPV, negative predictive value.*

**Table S3. Joint-level performance of visual evaluation of photos, by individual assessor**

| **Performance metric** | **Assessor** | | | |
| --- | --- | --- | --- | --- |
|  | **Assessor 1** | **Assessor 2** | **Assessor 3** | **Assessor 4** |
| **PPV** | 8/61 (0.13) | 12/74 (0.16) | 11/189 (0.06) | 9/40 (0.22) |
| **SEN** | 8/69 (0.12) | 12/69 (0.17) | 11/69 (0.16) | 9/69 (0.13) |
| **SPE** | 2998/3051 (0.98) | 2989/3051 (0.98) | 2873/3051 (0.94) | 3020/3051 (0.99) |
| **NPV** | 2998/3059 (0.98) | 2989/3046 (0.98) | 2873/2931 (0.98) | 3020/3080 (0.98) |

*PPV, positive predictive value; Sen, sensitivity; Spe, specificity; NPV, negative predictive value.*

**Table S4. Joint-level performance of visual evaluation of videos, by individual assessor**

| **Performance metric** | **Assessor** | | | |
| --- | --- | --- | --- | --- |
|  | **Assessor 1** | **Assessor 2** | **Assessor 3** | **Assessor 4** |
| **PPV** | 12/48 (0.25) | 26/55 (0.47) | 21/154 (0.14) | 11/24 (0.45) |
| **SEN** | 12/75 (0.16) | 26/75 (0.35) | 21/75 (0.28) | 11/75 (0.15) |
| **SPE** | 2889/2925 (0.99) | 2896/2925 (0.99) | 2792/2925 (0.95) | 2912/2925 (0.995) |
| **NPV** | 2889/2952 (0.98) | 2896/2945 (0.98) | 2792/2846 (0.98) | 2912/2976 (0.98) |

*PPV, positive predictive value; Sen, sensitivity; Spe, specificity; NPV, negative predictive value.*

**Table S5. Person-level pairwise agreement with Cohen’s Kappa for photos and videos**

| **Assessor pair** | **Photo** | **Video** |
| --- | --- | --- |
| *2,3* | 0.12 | 0.17 |
| *1,3* | 0.24 | 0.16 |
| *3,4* | 0.14 | 0.12 |
| *1,2* | 0.15 | 0.22 |
| *2,4* | 0.37 | 0.4 |
| *1,4* | 0.21 | 0.23 |

**Table S6. Joint-level pairwise agreement with Cohen’s Kappa for photos and videos**

| **Assessor pair** | **Photo** | **Video** |
| --- | --- | --- |
| *2,3* | 0.15 | 0.21 |
| *1,3* | 0.12 | 0.16 |
| *3,4* | 0.08 | 0.10 |
| *1,2* | 0.11 | 0.24 |
| *2,4* | 0.20 | 0.27 |
| *1,4* | 0.04 | 0.13 |

| 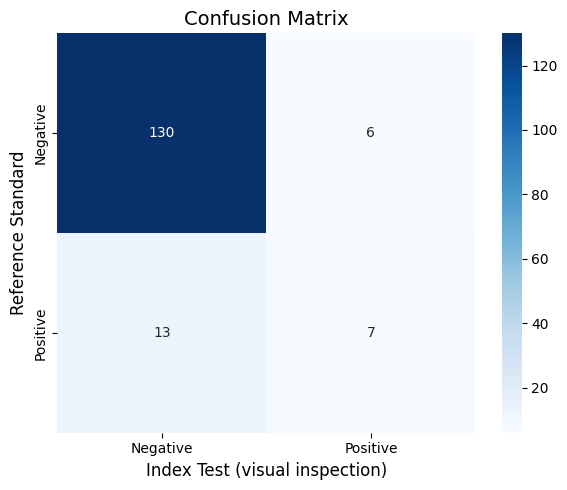  a) Person-Level Image Confusion Matrix |
| --- |
| 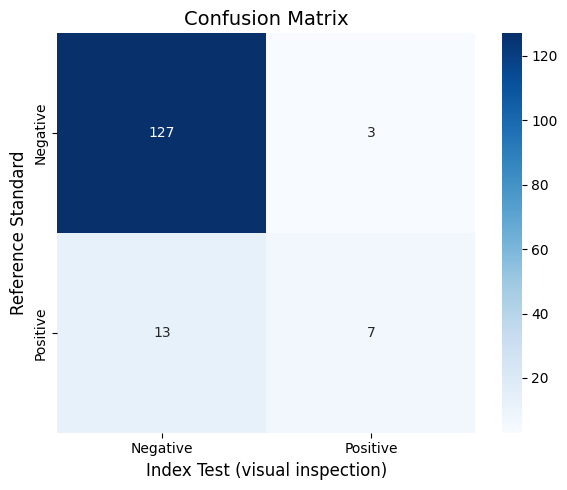  b) Person-Level Video Confusion Matrix |
| 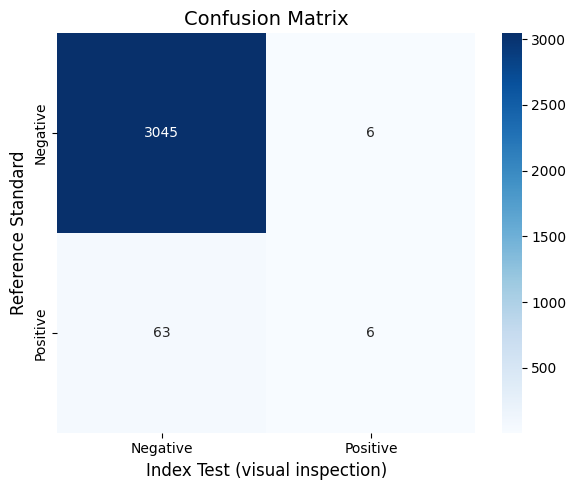  c) Joint-Level Image Confusion Matrix |
| 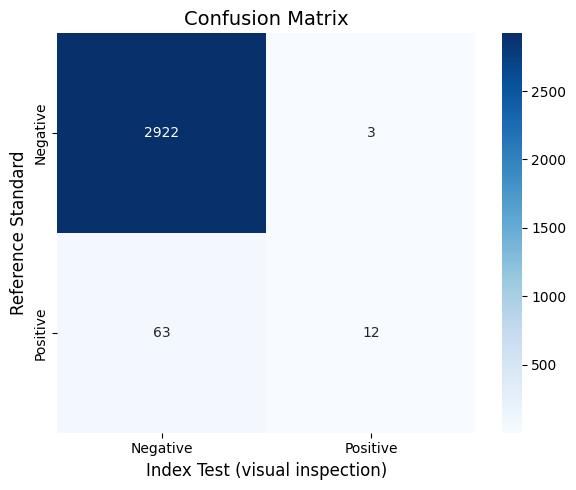  d) Joint-Level Video Confusion Matrix |

**Figure S1. Confusion Matrices**

*Index test reflects the overall assessor performance, using a threshold of ≥3/4 assessors labeling positive for joint effusion being considered a positive index test.*
